# The Utilization of a Fiberglass Mesh–Reinforced Foamcrete Jacketing System to Enhance Mechanical Properties

**DOI:** 10.3390/ma15175825

**Published:** 2022-08-24

**Authors:** Anisah Mat Serudin, Md Azree Othuman Mydin, Mohd Nasrun Mohd Nawi, Rafikullah Deraman, Marti Widya Sari, Mohammad Firdaus Abu Hashim

**Affiliations:** 1 School of Housing, Building and Planning, Universiti Sains Malaysia, Minden 11800, Penang, Malaysia; 2 Disaster Management Institute (DMI), School of Technology Management and Logistics, Universiti Utara Malaysia, Sintok 06010, Kedah, Malaysia; 3 Jamilus Research Centre, Faculty of Civil Engineering and Built Environment, Universiti Tun Hussein Onn Malaysia, Batu 86400, Pahat, Malaysia; 4 Faculty of Science and Technology, Universitas PGRI Yogyakarta, Jl. PGRI I No. 117, Sonosewu, Yogyakarta 55182, Indonesia; 5 Center of Excellence Geopolymer and Green Technology (CEGeoGTech), Faculty of Mechanical Engineering Technology, Universiti Malaysia Perlis, Kampus Tetap Pauh Putra, Arau 02600, Perlis, Malaysia

**Keywords:** foamcrete, compressive, tensile, flexural, failure modes, concrete behavior

## Abstract

Foamcrete is fabricated by combining mortar slurry and constant foam. Owing to the existence of air entrained in its cementitious matrix, foamcrete is tremendously brittle compared to normal-strength concrete. The addition of synthetic and natural plant fibers demonstrates an enhancement to foamcrete’s mechanical performance yet exerts a harmful effect on long-term performance. Depreciation of natural plant fibers and corrosion of synthetic fibers impact the lifespan and durability properties of foamcrete. Hence, this study aims to investigate the mechanical properties and mode of failures of foamcrete reinforced with fiberglass mesh (FM). The parameters assessed were the compression, flexural, and splitting tensile strengths of 1100 kg/m^3^ density foamcrete confined with various layers of 145 g/m^2^ of FM. The optimal foamcrete mechanical properties enhancement was attained with three-layer jacketing. Notable augmentations of 108% in the compressive strength, 254% in flexural strength, and 349% in splitting tensile strength were achieved in comparison to the control specimens at day 28. The control foamcrete samples under compressive, flexural, and tensile loads encountered brittle failure in comparison to the confined foamcrete. The mode of failure under the tensile load indicates that only a slight crack occurred at the upper side and a perpendicular mark at the lateral section of the foamcrete with one to three layers of FM jacketing. Thus, the jacketing system of foamcrete with FM enhances the behavior and load carrying capacity of foamcrete to the extent of preventing the propagation of cracks.

## 1. Introduction

In the construction industry, concrete is recognized as a brittle material with low tensile strength [1]. However, due to its benefits as a structural element, much research has been carried out and new development products discovered to enhance its performance [2]. Thousands of years ago, people added straw to clay bricks to make buildings tougher. This idea evolved, and metallic reinforcement was invented, followed by conventional steel reinforcement, to improve the ductility and toughness of concrete [3,4].

Nevertheless, these materials faced the problem of corrosion, which led to the failure of the structures over the long term. After that, short fibers were introduced into concrete, with the main advantage being the production of ultra-ductile concrete [4,5]. Later, fiber-reinforced concrete (FRC) in yield reinforcing elements (e.g., bars, strips, sheets, etc.) was utilized for the reinforcement, strengthening, or seismic retrofitting of new or existing concrete and masonry structures [6,7,8]. In the 1990s, Germany produced textile-reinforced concrete (TRC) [9]. Confinement reinforcement is one of the methods for strengthening concrete structures in civil engineering work [10]. Among the advantages of this method are that it is lightweight and easy to construct, has a high strength-to-weight proportion, and increases the toughness and ductility of concrete structures [11]. Much research has been conducted to investigate the mechanical properties and durability of confinement-reinforced concrete. Three major materials are used for confinement in civil engineering works: (1) concrete-filled steel tubes (CFSTs), (2) fiber-reinforced polymers (FRPs), and (3) textile fabrics [12]. 

Foamcrete is a type of cellular concrete with air entrapped using a suitable foaming agent that has become progressively widespread because of its distinctive characteristics such as controlled reduced density, minimum utilization of aggregates, and exceptional thermal insulation [13]. Furthermore, the decrease in structural dead load due to its lowered density can assist to rein in the construction process. The cellular structure of foamcrete is created by combining appropriate foaming agents that can be either protein- or synthetic-based. By correctly regulating the foaming agent quantity, foamcrete with densities ranging from 500 kg/m^3^ to 1800 kg/m^3^ can be attained [14]. Since a lesser content of aggregate is employed in the fabrication of LFC, the total quantity of binder materials is normally higher than those used in normal-strength concrete. Foamcrete is suitable for use as a filling material in partition panels and is utilized extensively in the construction industry [15]. With the speedy growth of the industrialized building system, there is huge potential to embrace foamcrete for producing partition walls for buildings. One of the challenges for the utilization of foamcrete in building construction is its low strength due to its porous matrix. When foamcrete is applied to precast building components, it takes a longer time to reach the expected compressive strength for demoulding than does normal strength concrete [16,17,18]. Hence, a method to enhance foamcrete’s mechanical properties is extremely vital so that it can be utilized in various applications in building construction. 

According to Cibulka et al. [19], lightweight concrete can be specially reinforced using grid-shaped fibers also known as textile fabric. Yin et al. [20] stated that fine-grained concrete can be used to accomplish good bonding between the textile fabric and the matrix. Moreover, Dalal et al. [21] highlighted that fluid concrete with a maximum filler size of 2 mm is needed to provide sufficient binding and to allow the load of the matrix to be transferred to the reinforcement. Therefore, a combination of foamcrete (produced with fine aggregate or sand) and textile fabric can produce a lightweight and thin concrete element with a high load-bearing capacity. Nevertheless, Portal et al. [22] stated that textile-reinforced concrete (TRC) has been devised as a flexible high-performance material but has not received wide acceptance owing to the absence of some necessary design features. As mentioned by Gayathri et al. [23], many researchers have been working with the idea of improving the performance of concrete by developing high-performance fibers such as TRC. They also explained that TRC is a type of reinforced concrete in which the fabric cage is utilized as a tensile reinforcing material in the concrete. Reinforced concrete with high-strength textile fabrics (carbon and glass) has a greater residual load-bearing capacity and is thus able to maintain the integrity of a structure after exposure to the maximum applied load. Gayathri et al. also highlighted that the strength of concrete reinforced with carbon and glass textile fabrics is significantly greater compared to unreinforced concrete.

Moreover, Younis et al. [24] compared the bond characteristics of a fabric-reinforced cementitious matrix (FRCM) and of concrete using three different types of textile fabric materials (carbon, polyphenylene benzobisoxazole (PBO), and glass) with a different number of layers. The results showed that the PBO– and glass–FRCM bond failure was more brittle compared to the carbon FRCM. However, the PBO textile showed greater bonding with the concrete. Younis et al. explained that the bond capacity and failure modes were influenced by the number of layers and, indeed, the length of the bonds applied in the concrete. Thus, from this study it can be concluded that the higher the number of layers and bond length, the greater the bond between the textile fabric and the matrix, as was obtained by PBO with two layers of textile fabric and a bond length of 200 mm.

Although carbon textile has high tensile strength, there are disadvantages to using this material as reinforcement due to its high initial cost and poor adhesion properties, unlike basalt and AR-glass [25]. Even though basalt textile has good tensile strength and high failure strength compared to carbon as well as good resistance to chemical attacks, its volume and tensile strength will decrease over time as it reacts to the alkaline environment in the concrete [26]. This is because the silicon dioxide (SiO_2_) in the basalt textile will react with the alkali solution. Basalt textile is also suitable as a reinforcement material, but its strength capacity is still less than that of carbon and glass textiles [27]. There are two main benefits to using AR-glass textile as a reinforcement material, namely good adhesion ability and cost efficiency [28]. Previously, glass fabric was faced with the problem of degradation due to alkaline attacks. However, as reported by Butler et al. [29], an enhancement was carried out with the inclusion of 16–20% weight fractions of zirconium dioxide; these reduced the flow of hydroxide ions into the form of the glass, and thus, the additional breakdown of the highly alkaline concentrate solution could be significantly reduced. The alkaline attack can be reduced by coating the textile fabric and using a specific concrete mixture containing fewer alkali ions.

Therefore, there is a possibility of woven fiberglass mesh being utilized as a reinforcement material in foamcrete. The results of previous studies proved that textile fabrics can also be employed to significantly enhance the mechanical properties of foamcrete. Hence, this paper will present the mechanical properties and mode of failure of foamcrete confined with different layers of fiberglass mesh (FM) under compression, flexural, and tensile loads. 

## 2. Methodology

### 2.1. Materials

**Figure 1:**
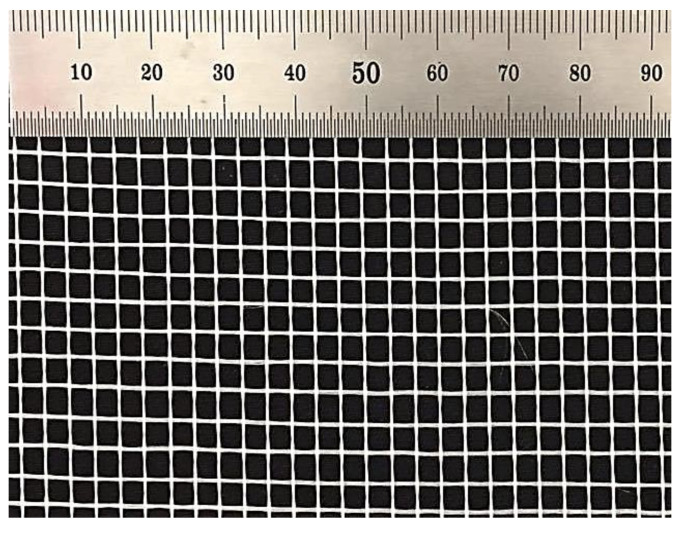
Fiberglass mesh (FM).

**Figure 2:**
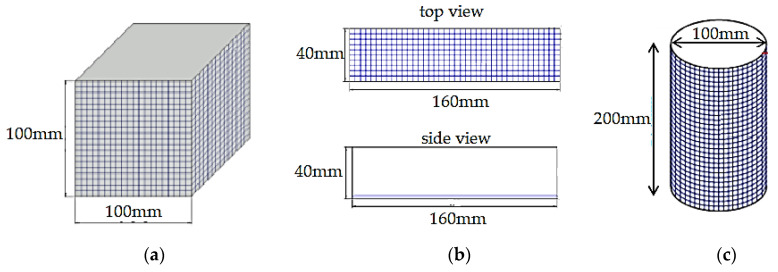
Position of the fibermesh confining foamcrete. (**a**) compression test (**b**) flexural test (**c**) tensile test.

The main materials utilized to fabricate the foamcrete included ordinary Portland cement (OPC), fine filler (sand), and distilled water. To generate a steady foam density, a foam generator was utilized. A protein-based foaming agent was utilized in this investigation. The density of the foam ranged between 75 and 85 g per litre. The fine filler (sand) employed is a natural aggregate in line with BS EN 12620 [30]. The specific gravity of the sand was 2.64. Moreover, the distilled water utilized for the preparation of the foamcrete was free from debris and unwanted organic substances. The water-to-cement proportion was maintained at 0.45 for the entire mixture, as it gave sufficient workability. Next, the fiberglass mesh (FM) employed in this study is categorized as a synthetic fiber (manmade fiber) and was chosen for its advantages as it is alkali-resistant and eco-friendly, has high strength, and requires no treatment; this is in contrast to carbon and basalt textiles, which require a coating regimen to improve their bonding with the cementitious matrix. The same can be said of natural fibers (coconut fibers, banana trunks, etc.), which need to be treated, for example, with sodium hydroxide (NaOH) solution to prolong their durability in foamcrete. The foamcrete specimen was confined with this textile fabric and placed in the matrix based on the specified test. Nevertheless, the specimen for the flexural test was not jacketed with this textile fabric but was laid in a longitudinal direction to the foamcrete specimen. In this investigation, 160 g (weight per area) of FM was utilized. Figure 1 shows the physical features of the FM, and its composition is detailed in Table 1. For this study, three discrete FM were employed: 1-layer, 2-layer, and 3-layer. Figure 1 shows the FM utilized in this study, in which the FM was positioned in the steel moulds before the foamcrete infilling process. Figure 2 shows the position of the FM confining foamcrete.

### 2.2. Mix Design

**Figure 3:**
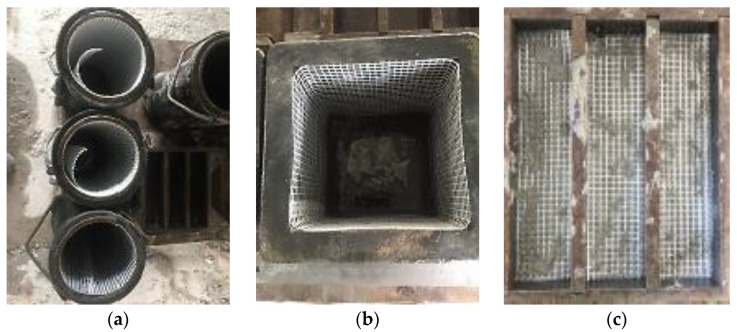
Fiberglass meshes were cut according to the size of the moulds. (**a**) cylinder (**b**) cube (**c**) prism.

The mix ratios for all the mixtures were fixed at 1:1.5 (cement–sand ratio) and 0.45 (water–cement ratio). Moreover, the density of the foamcrete was maintained at 1100 kg/m^3^. This was to ensure that comparable results were obtained for the confinement with FM as the main parameter to be investigated in this study. The control sample represents the foamcrete specimen without any confinement with FM, while the other specimens were confined with 1 to 3 layers of FM. Table 2 depicts the mix proportion prepared in this study. As shown in Figure 3, the fiberglass mesh was cut and laid according to the dimensions of the moulds before the foamcrete was inserted for the splitting tensile test (Figure 3a), the compression test (Figure 3b), and the flexural test (Figure 3c). 

### 2.3. Mechanical Property Testing

**Figure 4:**
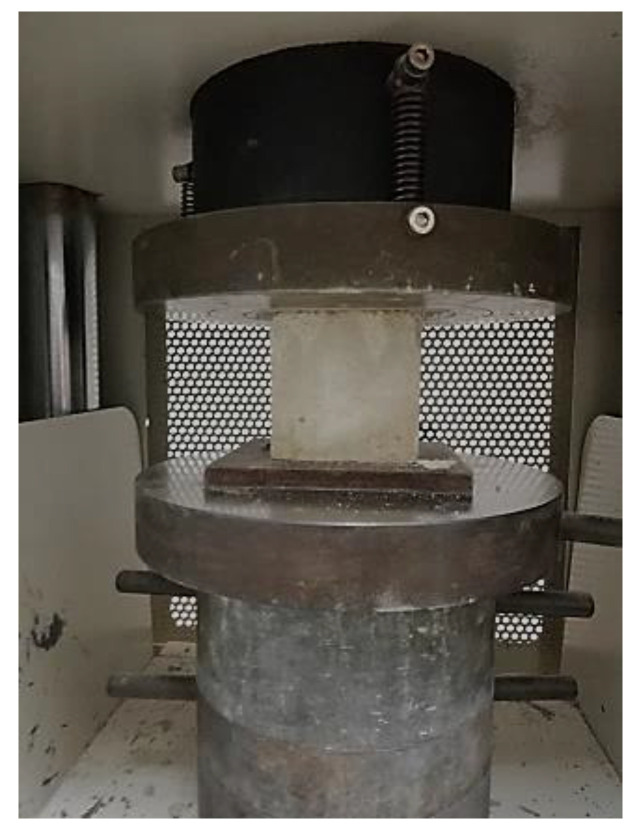
Setup for compression test.

Three mechanical tests were conducted to observe the mode of failures and the behavior of foamcrete specimens confined with various layers of FM under compression and flexural and tension load. The axial compressive strength test was carried out in line with BS EN 12390-3 [31] to analyze the failure mode of the foamcrete under a compression load with three cubic samples of 100 mm × 100 mm × 100 mm in size (Figure 4). The foamcrete compressive strength was established using the following formula:

Compressive strength = P/A(1)

where 

P = upper limit load incurred by the foamcrete specimen (N)

A = foamcrete specimen cross-sectional area (mm^2^)

**Figure 5:**
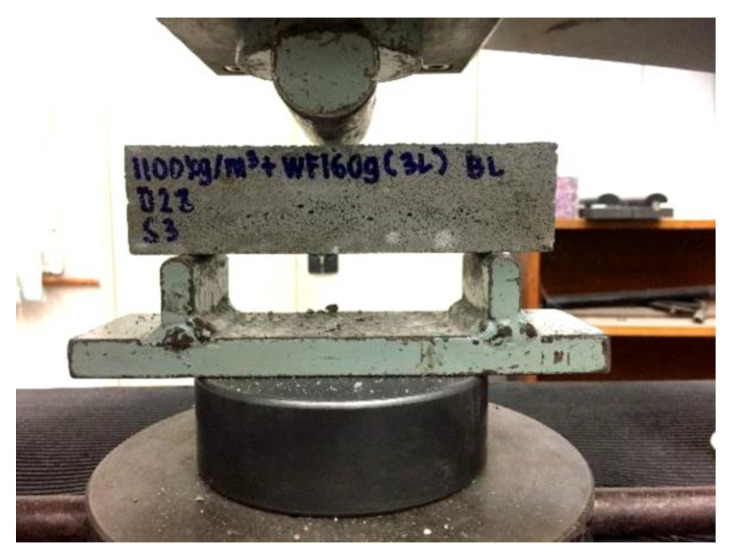
Setup for the flexural test.

Meanwhile, the flexural strength test was conducted using a Universal Flexural Device at a rate of 0.3 N/s to examine the failure mode of the foamcrete under a flexural load, as demonstrated in Figure 5. The flexural test was conducted via a three-point flexural method with a dimension of 40 mm × mm 40 × 160 mm, as prescribed in ASTM C348-19 [32]. The flexural strength of the foamcrete prism sample is governed using the following equation:

Flexural strength = 3PL/2bd^2^ (2)

where 

P = maximum load sustained by the foamcrete sample (N)

L = span length (mm)

b = width of tested prism (mm)

d = depth of tested prism (mm)

**Figure 6:**
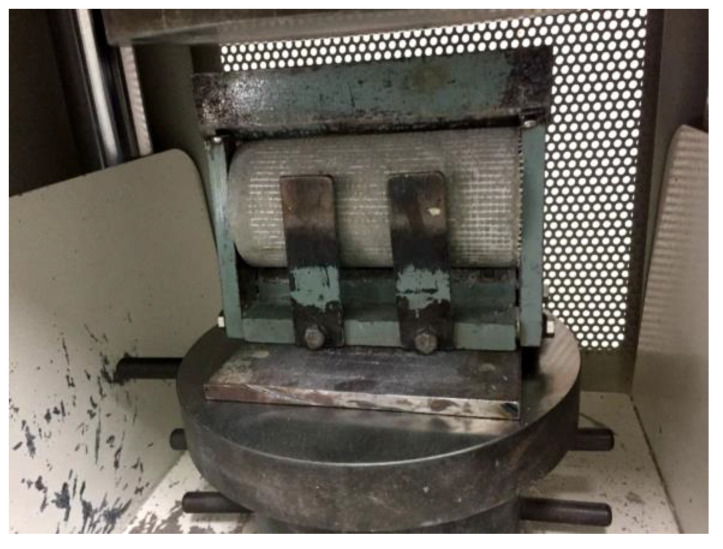
Setup for the splitting tensile test.

Next, to investigate the failure mode of the foamcrete under a tension load, the splitting tensile strength test was performed corresponding to BS EN 12390-6 [33]. A universal testing apparatus was used to perform the splitting tensile test with three cylindrical specimens measuring 100Ø mm × 200 mm, as indicated in Figure 6. The splitting tensile strength of the foamcrete was determined using the following formula:

Splitting tensile strength = 2P/ΠDL(3)

where 

P = the maximum load sustained by the foamcrete specimen (N)

D = the diameter of test cylinder (mm)

L = the length of the test cylinder (mm)

## 3. Results and Discussion

### 3.1. Compressive Strength

**Figure 7:**
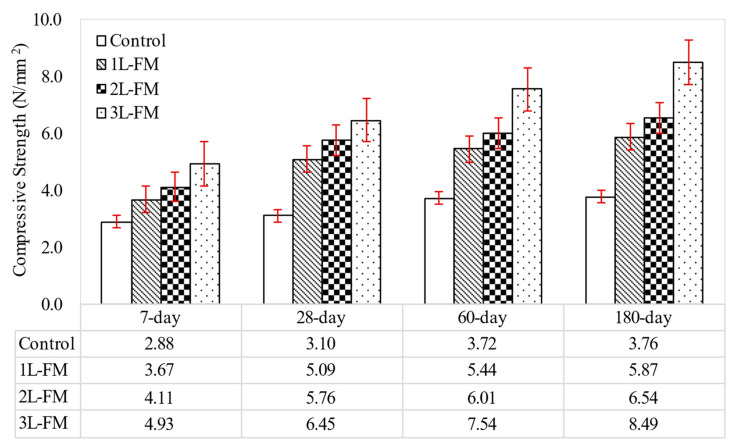
Compressive strength of foamcrete with different layers of FM.

Figure 7 reveals the present axial compressive strength results for foamcrete with densities of 1100 kg/m^3^ confined with different numbers of layers of FM. Figure 7 demonstrates the same pattern of strength growth, in which all the foamcrete mixes were enhanced with increasing curing time. Due to the brittleness of lightweight concrete, a reinforcing element is needed to boost its compressive strength [34]. Therefore, the confinement of foamcrete with the FM enhanced the compressive strength of the foamcrete. As can be observed, the confinement of foamcrete with 1 layer of FM boosted the compressive strength by 64% in comparison with the control foamcrete with the same density at day 28. Notable improvements of 86% were also obtained for the foamcrete specimens confined with two layers of FM. Furthermore, the highest increase in compressive strength found in this investigation was for the confinement of foamcrete with three layers of FM. The remarkable enhancements of 108% in compressive strength compared to the control at the respective densities proved that textile fabric has the potential to be utilized as a reinforcing element in foamcrete. All the enhancements achieved were due to the confinement with FM in the form of a jacket and the increase in the rigidity of the foamcrete. When the lateral deformation was developed because of the applied load, the tension in the jacket (FM confinement) was activated due to the lateral expansion of the foamcrete. In addition, the FM also worked to avoid microcracks and delay the expansion of microcracks on disclosure to a greater applied load. Enhanced resistance and ductility are controlled primarily by the fibers, which impede microcracks in the matrix. The good attachment between the textile fabric and the cementitious matrix is one of the justifications for improvement in the compressive strength of foamcrete. Moreover, Noor and Hazren [35] discovered that the number of layers of confinement in the concrete contributed to its compressive strength, where an enhancement of 54% was achieved by the application of between 1 to 2 layers of textile fabric. Huang et al. [36] also demonstrated that the use of textile fabric jackets improves the compressive behavior of plain concrete, and an increase in the number of textile fabric layers will lead to effective confinement due to the increase in the deformation capacity. Moreover, an improvement in the foamcrete load-bearing capacity leads to higher ultimate crack stress [37].

### 3.2. Flexural Strength

**Figure 8:**
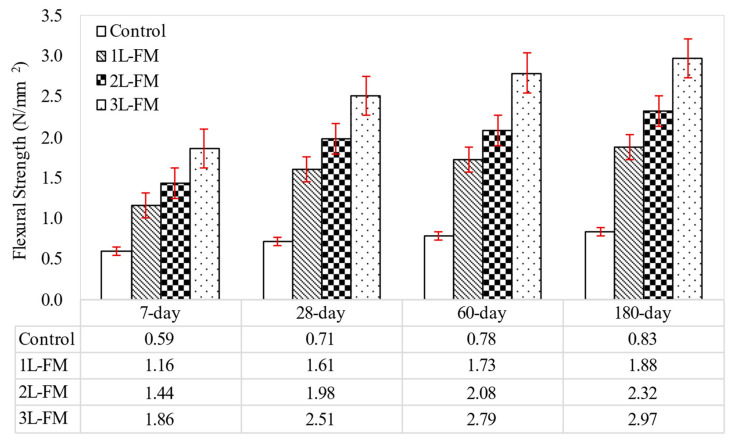
Flexural strength of foamcrete with different layers of FM.

Figure 8 reveals the flexural strength results for foamcrete with densities of 1100 kg/m^3^ confined with various layers of FM. The confined foamcrete was embedded with 1 layer, 2 layers, and 3 layers of FM placed 2 mm from the bottom layer, while the unconfined (control) specimens did not have any reinforcement in the tensile zone. Based on observations from these three figures, all the specimens gained flexural strength with increasing curing time. The strength development increased steadily for 180 days. It was noticed that the control specimens for the three respective densities displayed very poor flexural behavior. The control specimens had not been reinforced in the tensile zone, where the failure occurred suddenly as soon as a load was applied. A reinforcement strategy is needed to ensure that foamcrete can be utilized as a semi-structural or even more advanced structural element in construction work. In Figure 8, 1 layer of FM laid 2 mm from the bottom layer increased the flexural strength of the foamcrete by 127%, but when the number of layers was doubled (2 layers), the flexural strength was boosted by up to 179%, and it was effectively increased by 254% when 3 layers of textile fabric were added compared to the unreinforced specimens.

The structure of the FM itself was also the reason for the increase in the flexural strength of the foamcrete. A textile fabric is produced by combining several fibers to form a continuous fiber (known as a textile fabric) with a warp-and-weft structure, either in a coil or layered fashion [38]. The FM has a crimped geometrical structure, which provides enhanced bonding by mechanical effects. The further improvement in the attachment of the FM is due to mechanical affixing supplied by the fill yarns in the weft direction when the fabric is implanted in the foamcrete cementitious matrix. Moreover, the space between the fiber roving enables some sort of mechanical interlocking to occur between the FM and the matrix, which induces the strength of the foamcrete. This textile fabric is not only able to resist fractures because of sudden loading but is also capable of withstanding high fracture stiffness with superior impact strength. Reddy et al. [39] also claimed that the good bonding of glass fabric produces higher impact strength. Glass fiber is better in terms of both tensile strength and interfacial strength, resulting in the best impact strength compared to natural fibers (such as coir and palm fibers). Reddy et al. also highlighted that increasing the number of layers enhances the impact strength. This is because the total surface area of the textile fiber will increase so that further impact energy is dispersed between the fiber layers through extensive delamination.

### 3.3. Splitting Tensile Strength

**Figure 9:**
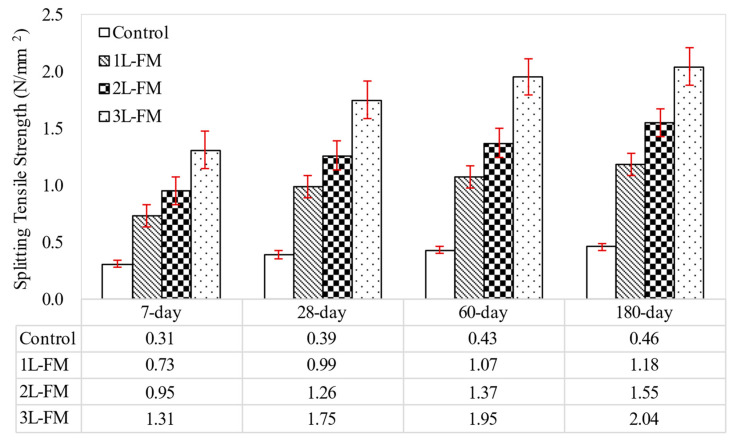
Splitting tensile strength of foamcrete with different layers of FM.

The factors that influence compressive strength equally affect the splitting tensile strength of foamcrete. Thus, unconfined foamcrete, which is brittle, possesses a lower tensile strength. This was proven by the results of the splitting tensile strength obtained in this study, which showed a similar trend as that of the compressive strength, as shown in Figure 9. The splitting tensile strengths of the control specimens were low due to the nonexistence of a strengthening element in the foamcrete matrix. Thus, the confinement of foamcrete with different numbers of layers of FM improved the performance of the foamcrete in terms of its tensile strength. When the foamcrete was wrapped in 1 layer of FM, the tensile strength increased by 154%. For the confinement with 2 layers of FM, the tensile strength increased by 223%. Superior augmentations of 349% in tensile strength were accomplished when the foamcrete specimens were confined with 3 layers of FM. Incredible augmentation of the splitting tensile strength in c proves that FM has the potential to be used as a reinforcing element in foamcrete. This performance also improved as the number of layers of textile fabric for the confinement increased. The FM had not only sturdy fiber-to-matrix attachment but also exceptionally durable fiber-to-fiber closeness, which permitted widening and prevented the foamcrete from collapsing. As highlighted by Parveen and Sharma [40], the enhancement of splitting tensile capacity is due to the holding ability of the fibers, which can aid in the splitting of the concrete. 

### 3.4. Failure Modes and Behavior under Compression Load

**Figure 10:**
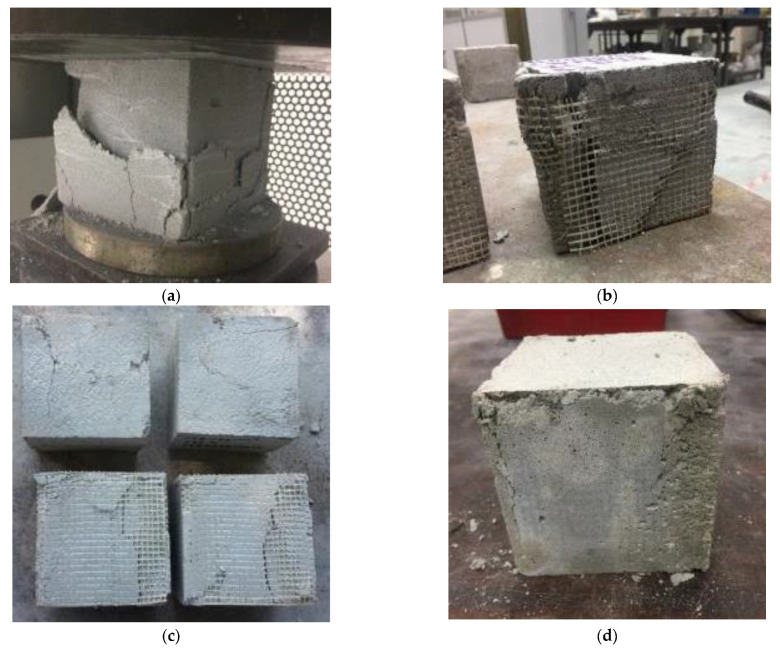
Failure modes of foamcrete under axial loading. (**a**) control specimen (**b**) 1 layer of FM (**c**) 2 layers of FM (**d**) 3 layers of FM.

Foamcrete possesses high porosity due to the addition of foam, which results in highly brittle behavior. Yankelevsky and Avnon [41] mentioned that a structural element must be able to resist numerous loadings and function appropriately in situations where it needs to be reinforced to achieve satisfactory ductility, shear resistance, etc. Figure 10 shows the results of this study’s investigation into the failure modes of the foamcrete specimens under an axial loading. For the control specimen (see Figure 10a), an obvious crack occurred approximately at the centre of the specimen. The initial crack began at the corner of the specimen and then propagated to the centre, where it formed a diagonal crack [42]. As the applied load increased, the outer surface of the concrete peeled and broke. When the foamcrete specimen was confined with 1 layer of FM, failure also occurred at the corner of the specimen, but the crack pattern was different compared to the unconfined foamcrete, as shown in Figure 10b. The crack pattern for this specimen appeared as a vertical crack located at the overlapping zone of the FM (corner of the specimen) [43]. The debonding of the FM happened when the applied load increased to its ultimate level. However, the addition of layers of FM for the confinement enhanced the failure modes of the foamcrete specimens. As presented in Figure 10c,d, although the foamcrete samples achieved their maximum loads, they persisted in good shape, where the FM acted as a cage and prevented a large explosion of the foamcrete. Therefore, this indicated that the confinement with FM improved the compressive strength of the foamcrete, and the number of layers applied significantly affected its performance. 

### 3.5. Failure Modes and Behavior under Flexural Load

**Figure 11:**
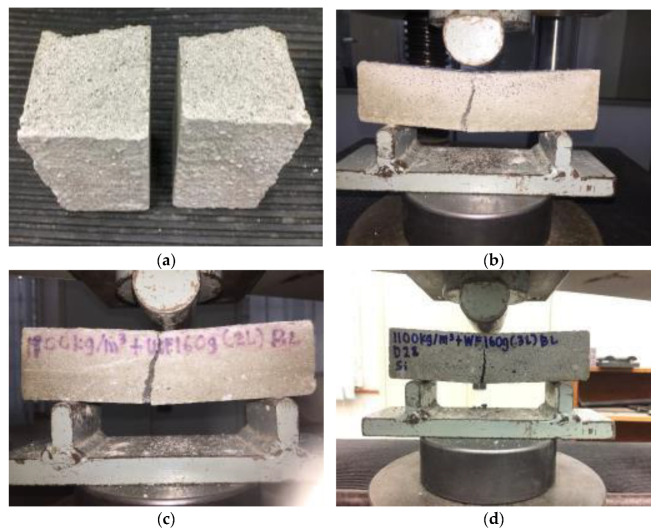
Failure modes of foamcrete under a flexural load. (**a**) Control specimen (**b**) 1 layer of FM (**c**) 2 layers of FM (**d**) 3 layers of FM.

Foamcrete is good when it comes to compression but weak with regard to tension, owing to the various microcracks triggered by the blend of delicate and brittle elements in the matrix. In this study, the failure modes of foamcrete subjected to a flexural load were examined and recorded, as shown in Figure 9. The control foamcrete faced sudden failure, where the specimen broke into two pieces as soon as the load was applied (refer to Figure 11a). Figure 11b–d shows the failure modes of the foamcrete confined with 1 layer, 2 layers, and 3 layers of FM, respectively. These three specimens encountered the same pattern of cracking, where a crack emerged at the bottom side and transmitted to the top section of the foamcrete specimen to form a vertical crack [44]. The confinement with FM improved the ductility and increased the flexural strength of the foamcrete. Moreover, the FM also provided an improved shear force by inhibiting the cracks from broadening due to the flexural stress. The number of layers of FM also influenced the performance of the foamcrete; it can be concluded that the higher the number of layers applied, the higher the degree of confinement achieved. 

### 3.6. Failure Modes and Behavior under Tensile Load

**Figure 12:**
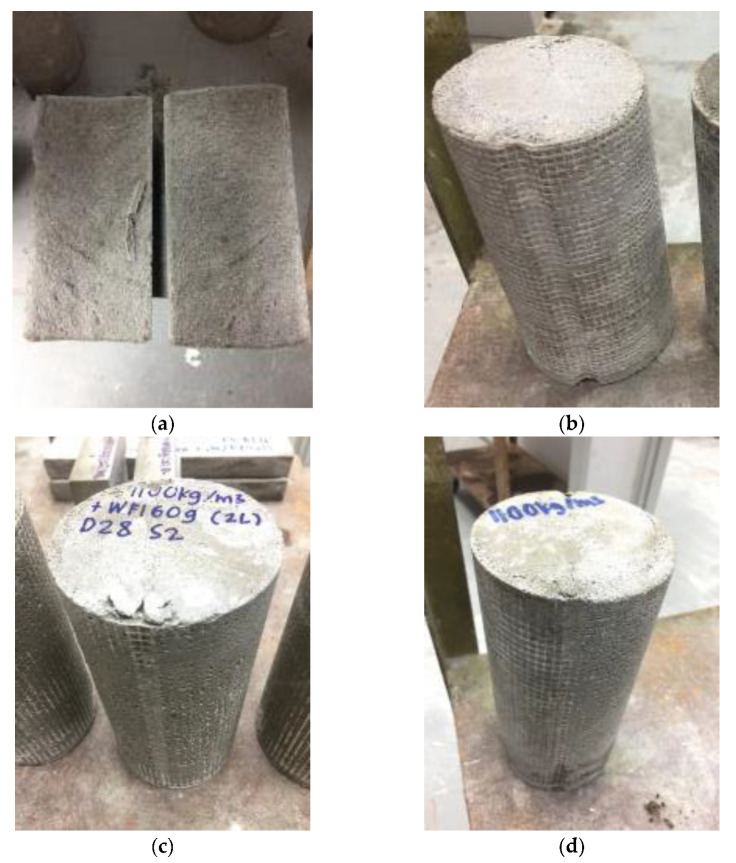
Failure modes of foamcrete under tension loading. (**a**) Control specimen (**b**) 1 layer of FM (**c**) 2 layers of FM (**d**) 3 layers of FM.

Figure 12 shows the failure modes of foamcrete under tension loading. When the control specimen reached its maximum applied load during testing, it was broken into two pieces by a vertical crack (see Figure 12a). This was caused by the brittleness of the foamcrete, which failed to resist the crack propagation. For the confined foamcrete, as shown in Figure 12b–d, small microcracks emerged at the upper part of the foamcrete and a perpendicular mark emerged at the lateral plane. The specimens did not break into two even after the failure due to the confinement with FM, which arrested the occurrence of a major crack. Therefore, this supports our previous statement that the technique of jacketing the foamcrete in FM increased its tensile strength compared to the unconfined specimens. 

### 3.7. Correlation between Foamcrete Compressive and Flexural Strengths

**Figure 13:**
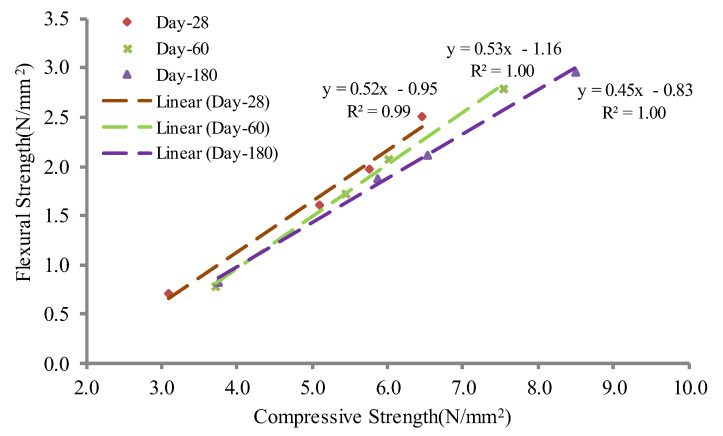
Correlation between foamcrete compressive and flexural strengths.

Figure 13 represents the correlation between the foamcrete compressive strength and flexural strength confined with 1–3 layers of FM. The compressive and flexural strengths of the foamcrete revealed a comparable tendency of enhancement. However, the flexural strength of the foamcrete was lower than its compressive strength due to its good performance with compression but weak behavior with tension. Thus, the confinement with woven fiberglass mesh in this study increased both the compressive and flexural strengths of the foamcrete. Moreover, the R^2^ obtained proved that there was a significant relationship between these two parameters, where all the R^2^ values were close to 1. In general, the development of compressive strength shared the same trend and retained a linear relationship with the development of flexural strength due to the confinement with woven fiberglass mesh, which improved the ductility, shear capacity, and energy absorption of the foamcrete. Besides, the number of layers of confinement also contributed to the high compressive and flexural strengths of the foamcrete. The flexural strengths were in the range of 58–60%, corresponding to the compressive strengths. 

### 3.8. Correlation between Foamcrete Flexural and Splitting Tensile Strengths

**Figure 14:**
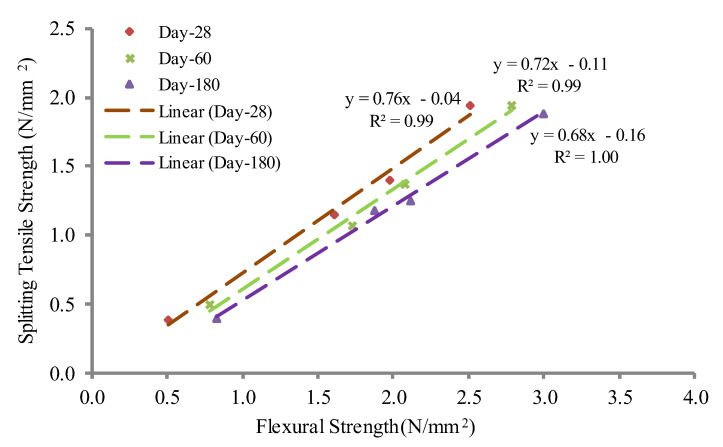
Correlation between foamcrete flexural and splitting tensile strengths.

The mechanical properties of concrete such as shear resistance, bond strength, and resistance to cracking depend on its tensile strength. Compared to the relationship between compressive strength and flexural strength, the correlation between tensile and flexural strengths showed a significant linear relationship, where the R^2^ value was equal to 1, as shown in Figure 14. This indicated that the increase in the flexural strength of the foamcrete was parallel with the increase in the tensile strength. This phenomenon was due to the confinement with woven fiberglass mesh laid longitudinally to the applied load, which prevented the sudden failure of the foamcrete and acted as a bridge to prevent the specimen from separating into two pieces. Longitudinal fiber laminates always contribute to a higher impact strength and a more efficient dissipation of impact energy. The tensile strength values were in the range of 74 to 76%, corresponding to the flexural strength values. 

## 4. Conclusions

In this investigation, the mechanical properties of foamcrete confined with various layers (1–3 layers) of fiberglass mesh (FM) were investigated, including compressive, flexural, and splitting tensile strengths. Additionally, the failure modes of foamcrete were observed. Based on the experimental investigation, the following conclusions are drawn.

1. For compressive strength, the jacketing of foamcrete with 1 layer of FM enhanced the strength by 64% compared to the control foamcrete at day 28. Significant enhancements of 86% were also achieved with 2-layer confinement of FM. Additionally, the highest upsurge of compressive strength was attained with 3-layer jacketing of FM. The notable rise of 108% in the compressive strength compared to the control sample at day 28 demonstrated that FM has the possibility to be employed as a strengthening element in foamcrete. The enrichments that were attained were due to the confinement with FM in the form of a jacket, which improves the foamcrete rigidity.
2. As far as flexural strength is concerned, the strength development increased steadily for 180 days. The control foamcrete samples which were not reinforced in the tensile zone displayed poor flexural behavior; they experienced sudden failure as soon as a load was applied. Reinforcement with 1 layer of FM laid 2 mm from the bottom layer augmented the foamcrete flexural strength by 127%. When the number of layers was doubled (2 layers), the flexural strength was improved by up to 179%. Flexural strength was excellently augmented by 254% when 3 layers of FM were attached compared to the unreinforced foamcrete samples.
3. For splitting tensile strength, the control foamcrete attained low tensile properties due to the nonexistence of a fortifying component in the foamcrete matrix. When the foamcrete was wrapped in 1 layer of FM, the splitting tensile strength improved by 154%. For the jacketing with 2 layers of FM, the enhancement was nearly 223%. Outstanding augmentations of 349% in tensile strength were achieved when the foamcrete was confined with 3 layers of FM.
4. All the unreinforced foamcrete showed sudden failure as soon as the load was applied (under compression, flexural, and splitting tensile loads). This was due to the brittleness of the foamcrete specimens, which caused them to crack easily when exposed to a higher applied load. Meanwhile, the reinforced foamcrete (1–3 layers) showed a higher ductility, where the FM delayed the occurrence of crack propagation. The FM also prevented the foamcrete specimens from separating into two pieces, and it managed to hold the cementitious matrix even when the maximum load was applied.

## Figures and Tables

**Table 1:** Chemical composition of FM.

Oxide Components	Percentage by Weight (%)
SiO_2_	65.5
ZrO_2_	17.3
TiO_2_	1.2
Al_2_O_3_	1.4
Fe_2_O_3_	1.9
CaO	6.9
MgO	0.8
Na_2_O	0.7
K_2_O	0.4
B_2_O_3_	2.2
Li_2_O	0.3
F_2_	0.5
Others	0.9

**Table 2:** Foamcrete mix design.

Sample Coding	Foamcrete Density (kg/m^3^)	FM	OPC (kg)	Fine Sand (kg)	Distilled Water (kg)
1100-CTRL	1100	-	44.96	67.44	20.23
1L-FM-1100	1100	1 layer	44.96	67.44	20.23
2L-FM-1100	1100	2 layers	44.96	67.44	20.23
3L-FM-1100	1100	3 layers	44.96	67.44	20.23

## Data Availability

Not applicable.

## References

[1] Decký M., Drusa M., Zgútová K., Blaško M., Hájek M., Scherfel W. (2016). Foam Concrete as New Material in Road Constructions. Procedia Eng..

[2] Wu J., Lv C., Pi R., Zhang H., Bi Y., Song X., Wang Z. (2021). The stability and durability of silt-based foamed concrete: A new type of road engineering material. Constr. Build. Mater..

[3] Mohamad N., Iman M.A., Mydin M.A.O., Samad A.A.A., Rosli J.A., Noorwirdawati A. (2018). Mechanical properties and flexure behaviour of lightweight foamed concrete incorporating coir fibre. IOP Conf. Ser. Earth Environ. Sci..

[4] Serri E., Othuman Mydin M.A., Suleiman M.Z. (2014). The effects of oil palm shell aggregate shape on the thermal properties and density of concrete. Adv. Mat. Res..

[5] Sui L., Luo M., Yu K., Xing F., Li P., Zhou Y., Chen C. (2018). Effect of engineered cementitious composite on the bond behavior between fiber-reinforced polymer and concrete. Compos. Struct..

[6] Kong K., Mesticou Z., Michel M., Larbi A.S., Junes A. (2017). Comparative characterization of the durability behaviour of textile reinforced concrete (TRC) under tension and bending. Compos. Struct..

[7] Butler M., Hempel S., Mechtcherine V. (2011). Modelling of ageing effects on crack-bridging behaviour of AR-glass multifilament yarns embedded in cement-based matrix. Cem. Concr. Res..

[8] Portal N.W., Perez I.F., Thrane L.N., Lundgren K. (2014). Pull-out of textile reinforcement in concrete. Constr. Build. Mater..

[9] Valeri P., Ruiz M.F., Muttoni A. (2020). Modelling of Textile Reinforced Concrete in bending and shear with Elastic-Cracked Stress Fields. Eng. Struct..

[10] Saidi M., Gabor A. (2020). Experimental analysis of the tensile behaviour of textile reinforced cementitious matrix composites using distributed fibre optic sensing (DFOS) technology. Constr. Build. Mater..

[11] Liu S., Rawat P., Wang X., Zhu D. (2019). Low velocity impact behavior of AR-glass textile reinforced mortar under varying range of loading and temperatures. Constr. Build. Mater..

[12] Tlaiji T., Vu X.H., Ferrier E., Larbi A.S. (2018). Thermomechanical behaviour and residual properties of textile reinforced concrete (TRC) subjected to elevated and high temperature loading: Experimental and comparative study. Compos. Part B Eng..

[13] Mohd Zamzani N., Othuman Mydin M.A., Abdul Ghani A.N. (2019). Mathematical regression models for prediction of durability properties of foamed concrete with the inclusion of coir fibre. Int. J. Eng. Adv..

[14] Amran Y.H.M., Farzadnia N., Abang Ali A.A. (2015). Properties and applications of foamed concrete; a review. Constr. Build. Mater..

[15] Mydin M.A.O., Musa M., Ghani A.N.A. (2018). Fiber glass strip laminates strengthened lightweight foamed concrete: Performance index, failure modes and microscopy analysis. AIP Conf. Proc..

[16] Serudin A.M., Othuman M.A.M., Ghani A.N.A. (2021). Effect of lightweight foamed concrete confinement with woven fiberglass mesh on its drying shrinkage. Rev. Ing. Construcción.

[17] Kunhanandan Nambiar E.K., Ramamurthy K. (2006). Influence of filler type on the properties of foam concrete. Cem. Concr. Compos..

[18] Nensok M.H., Mydin M.A.O., Awang H. (2021). Investigation of Thermal, Mechanical and Transport Properties of Ultra Lightweight Foamed Concrete (ULFC) Strengthened with Alkali Treated Banana Fibre. J. Adv. Res. Fluid Mech. Therm. Sci..

[19] Cibulka T., Musil L., Vodička J. (2019). The Lightweight Textile Reinforced Concrete for Thin-Walled Structures. Acta Polytech. CTU Proc..

[20] Yin S., Wang B., Wang F., Xu S. (2017). Bond investigation of hybrid textile with self-compacting fine-grain concrete. J. Ind. Text..

[21] Dalal M., Goumairi O., El Malik A. (2017). Study of the internal confinement of concrete reinforced (in civil engineering) with woven reinforcement. IOP Conf. Ser. Mater. Sci. Eng..

[22] Portal N.W., Lundgren K., Wallbaum H., Malaga K. (2015). Sustainable potential of textile-reinforced concrete. J. Mater. Civ. Eng..

[23] Gayathri C.N., Singh R.B., Dhanalakshmi G. (2018). Mechanical behaviour of textile reinforced concrete. Int. Res. J. Eng. Technol. (IRJET).

[24] Younis A., Ebead U., Shrestha K. (2020). Tensile characterization of multi-ply fabric-reinforced cementitious matrix strengthening systems. Struct. Concr..

[25] Serudin A.M., Mydin M.A.O., Ghani A.N.A. (2021). Influence of Fibreglass Mesh on Physical Properties of Lightweight Foamcrete. IIUM Eng. J..

[26] Yin S., Jing L., Yin M., Wang B. (2018). Mechanical properties of textile reinforced concrete under chloride wet-dry and freeze-thaw cycle environments. Cem. Concr. Compos..

[27] Machovec J., Reiterman P. (2018). Influence of Aggressive Environment on the Tensile Properties of Textile Reinforced Concrete. Acta Polytech..

[28] Goldfeld Y., Perry G. (2018). AR-glass/carbon-based textile-reinforced concrete elements for detecting water infiltration within cracked zones. Struct. Health Monit..

[29] Butler M., Mechtcherine V., Hempel S. (2010). Durability of textile reinforced concrete made with AR glass fibre: Effect of the matrix composition. Mater. Struct..

[30] (2013). Aggregates for Concrete.

[31] (2011). Testing Hardened Concrete. Compressive Strength of Test Specimens.

[32] (2019). Standard Test Method for Flexural Strength of Hydraulic-Cement Mortars.

[33] (2009). Testing Hardened Concrete. Tensile Splitting Strength of Test Specimens.

[34] Shah S.P., Naaman A.E., Moreno J. (1983). Effect of confinement on the ductility of lightweight. Int. J. Cem. Compos. Lightweight Concr..

[35] Noor N.A., Hazren M. Fibre Reinforced Polymer Concrete Structures—Opportunities and Concerns. Proceedings of the International Conference on Building Science and Engineering.

[36] Huang L., Yang X., Yan L., He K., Li H., Du Y. (2016). Experimental study of polyester fiber-reinforced polymer confined concrete cylinders. Text. Res. J..

[37] Kearsley E.P., Wainwrightb P.J. (2001). The effect of high fly ash content on the compressive strength of foamed concrete. Cem. Concr. Res..

[38] Vogel F., Holcapek O., Konvalinka P. (2016). Response of high-performance fibre reinforced concrete reinforced by textile reinforcement to impact loading. Acta Polytech..

[39] Reddy G.V., Naidu S.V., Rani T.S. (2008). Kapok/glass polyester hybrid composites: Tensile and hardness properties. J. Reinf. Plast. Compos..

[40] Parveen A.S., Sharma A. (2013). Structural behaviour of fibrous concrete using polypropylene fibres. Int. J. Mod. Eng. Res..

[41] Yankelevsky D.Z., Avnon I. (1998). Autoclaved aerated concrete behavior under explosive action. Constr. Build. Mater..

[42] Othuman Mydin M.A., Sahidun N.S., Mohd Yusof M.Y., Noordin N.M. (2015). Compressive, flexural and splitting tensile strengths of lightweight foamed concrete with inclusion of steel fibre. J. Teknol..

[43] Jitchaiyaphum K., Sinsiri T., Jaturapitakkul C., Chindaprasirt P. (2013). Cellular lightweight concrete containing high-calcium fly ash and natural zeolite. Int. J. Miner. Met. Mater..

[44] Musa M., Othuman Mydin M.A., Abdul Ghani A.N. (2019). Influence of oil palm empty fruit bunch (EFB) fibre on drying shrinkage in restrained lightweight foamed mortar. Int. J. Innov. Technol. Exp. Eng..

